# Forward Osmosis for Metal Processing Effluents under Similar Osmotic Pressure Gradients

**DOI:** 10.3390/membranes13050501

**Published:** 2023-05-10

**Authors:** Noel Devaere, Vladimiros Papangelakis

**Affiliations:** Department of Chemical Engineering and Applied Chemistry, University of Toronto, 200 College Street, Toronto, ON M5S 3E5, Canada; noel.devaere@mail.utoronto.ca

**Keywords:** brines, effluent treatment, forward osmosis, membranes, mine water, osmotic gradient

## Abstract

Water recovery from aqueous effluents in the mining and metals processing industry poses a unique challenge due to the high concentration of dissolved salts typically requiring energy intensive methods of treatment. Forward osmosis (FO) is a lower energy technology which employs a draw solution to osmotically extract water through a semi-permeable membrane further concentrating any feed. Successful FO operation relies on using a draw solution of higher osmotic pressure than the feed to extract water while minimizing concentration polarization to maximize the water flux. Previous studies employing FO on industrial feed samples commonly used concentration instead of osmotic pressures for feed and draw characterization; this led to misleading conclusions on the impact of design variables on water flux performance. By employing a factorial design of experiments methodology, this study examined the independent and interactive effects on water flux by: osmotic pressure gradient, crossflow velocity, draw salt type, and membrane orientation. With a commercial FO membrane, this work tested a solvent extraction raffinate and a mine water effluent sample to demonstrate application significance. By optimizing with osmotic gradient independent variables, water flux can be improved by over 30% without increasing energy costs or compromising the 95–99% salt rejection of the membrane.

## 1. Introduction

Water use in the mining and metals industry is of concern from both the environmental and financial aspects. Data from the industrial water survey (Statistics Canada) show that Canadian mining and metals annual water intake and discharge was 1.6 billion m^3^, in 2020 [1,2]. Additionally, many of these wastes were in remote locations, such as Canada’s North, where access to energy is limited and commonly from fossil fuels. Consequently, to tackle the intake and effluent problem, there is a continuing need to identify low-energy water recycling solutions which can treat the liquid wastes found in mining and metals industries. 

These liquid streams are mixtures of concentrated dissolved inorganic salts which quite often need to be dewatered to recover process water for recycling and concentrated further for waste treatment. Because of their high total dissolved solids (TDS), these aqueous streams do not respond well to most conventional treatment systems. For example, the separation of water from solutions of alkali and alkali earth metal ions (Na^+^, Mg^2+^, Ca^2+^) in chloride or sulphate solutions is typically carried out via reverse osmosis (RO) or multi-stage flash distillation (MSFD) [3]. RO fails in many applications where the dissolved ion concentration exceeds that of seawater mainly due to the irreversible fouling that occurs from precipitation of contaminants such as gypsum (CaSO_4_•2H_2_O) [4,5]. MSFD is cost prohibitive due to the high thermal energy cost even with good heat recovery [6].

Forward osmosis (FO) is a spontaneous osmotically driven membrane process which relies solely on an osmotic pressure gradient as opposed to a physical pressure gradient to drive water removal. In FO, there are two aqueous solutions to consider: a feed solution from which water is being removed and a concentrated draw solution on the opposite side of a semi-permeable membrane. The key to FO operation is that the draw solution must have a higher osmotic pressure than the feed solution because water moves from a low osmotic pressure to high osmotic pressure. The difference in osmotic pressures is referred to as an osmotic gradient. Driven by the osmotic gradient, water from the feed solution is drawn spontaneously through the membrane, and the salts are rejected which results in a more concentrated feed. The concentrated feed is at a higher osmotic pressure, and the diluted draw solution is at a lower osmotic pressure. The reduced volume feed can be sent to either further processing or for disposal. 

In a continuous operation, clean water must be recovered from the draw solution in a separate second step to reconcentrate the draw solution. Depending on the type of the draw solute, different reconcentration methods are available such as thermal decomposition of thermolytic salts such as ammonium or trimethylamine (TMA) carbonate [7]. Previous work by Kolliopoulos et al. [6] on energy calculations showed that FO with thermolytic TMA carbonate decomposition is indeed a low energy alternative to MSFD. While it is well established that a commercial FO process will always use more energy than a commercial RO process, with FO, there is potential for the energy to be supplied from waste sources or the natural environment [8]. A novel process proposed recently by our group [9] involves the separation of water from the dilute draw solution as ice by freezing and/or by harnessing the cold winters in polar regions [10,11]. The draw solution reconstitution to its initial concentration is beyond the scope of this paper, but it must be noted that it represents the savings of the energy-intensive part of the operation.

**Figure 1:**
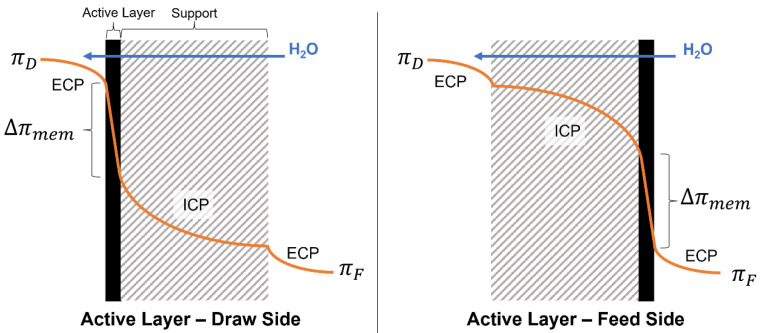
Osmotic pressure (orange curve) variation from the feed ($\pi_{F}$) to the draw ($\pi_{D}$) side of the membrane due to ECP and ICP in both membrane orientations: active layer–draw side (AL-DS) and active layer–feed side (AL-FS). The direction of water flow is indicated by the blue arrow [14].

A gradient in the chemical potential of water is the driving force for FO, which is reflected by the osmotic pressure (π) gradient. However, bulk osmotic pressure gradient between the feed and draw solutions alone cannot be used as a performance predictor of an FO membrane because of concentration polarization (CP), which, in turn, results in lowering the real driving force for water separation. CP refers to changes in the water chemical potential due to concentration changes within the boundary layers at both membrane interfaces. Commercial FO membranes consist of two layers: a dense active layer responsible for the separation of water from dissolved ions and a porous support layer which improves the mechanical strength of the membrane. Two orientations are possible: active layer–draw side (AL-DS) and active layer–feed side (AL-FS), as shown in Figure 1. CP arises from mass transfer gradients developing at the boundary layers which decrease the bulk osmotic gradient (Δπ = $\pi_{D}$ − $\pi_{F}$) to an effective $\Delta \pi_{mem}$ value across the membrane (see Figure 1). There are two types of CP: internal (ICP) and external (ECP). Mass transfer limitations of solutes inside the porous support layer result in ICP. Mass transfer limitations from the bulk to the surface of the active layer and support layers result in ECP [12,13], as shown in Figure 1.

The ECP on the feed side causes an increase in concentration at the membrane-feed solution interface which can saturate dissolved components, and result in local precipitation leading to membrane fouling [15]. In any membrane process, this fouling lowers water flux, and thus, it requires cleaning. However, cleaning can never reverse the membrane to its initial state leading to irreversible fouling and eventual membrane failure [4]. In FO, lower water flux due to fouling decreases the extent of ICP which essentially increases the real the osmotic gradient at the membrane which further increases the water flux. This trade-off between ICP and fouling is called the ICP self-compensation effect [16,17], and it improves the resilience and stability of FO in high fouling feeds; so, FO operation remains less impacted by irreversible fouling. Since RO does not benefit from such effects [16], irreversible fouling remains a costly drawback to RO technology [15], and it makes FO a promising option for dewatering concentrated inorganic, fouling-prone feeds.

While there is an abundance of literature on FO [18,19], aqueous streams in the mining and metals industries received limited attention. The most relevant study to this work is by Dou et al. [20], who successfully used FO to concentrate a vanadium-containing leach solution. In another example, Pramanik et al. [21] reviewed the rejection of rare earth elements in simulated acid mine drainage using FO to concentrate the stream. Another waste, similar to that found in the mining and metals industry though more dilute, is landfill leachate, which operated at full-scale successfully with an FO system to recover water before a further RO step produced clean water [13]. In another study, the use of FO was studied in setups where the dilutive and concentrative effects were desired in two separate parts of a plant, such as in printed circuit board manufacturing [22]. However, a majority of case studies in FO dealt with dilute solution concentrations unlike those found in typical mining and metals industry operations which are often much greater than that of seawater (35,000 ppm) [7].

The water removal performance is affected by operating variables such as: feed and draw solution electrolyte diffusivities, feed and draw crossflow velocities, and the orientation of the membrane [12,14]. In many studies dealing with FO operating variables on real or synthetic feeds [20,21,22,23,24,25,26,27,28,29,30,31], concentration, or a proxy for concentration (permeate volume, concentration factor), was used instead of osmotic pressure gradient which obfuscates the impact of variable changes with osmotic pressure changes on kinetics. In the few cases where the osmotic pressure gradient was used [32,33,34] with real feeds, few operating variables were investigated, and they tested low osmotic pressure feeds (less than 20 bar). To assess the impact of an operating variable on FO performance for any feed, it is necessary to evaluate the above operating variables under similar bulk osmotic pressure gradients, or else it is impossible to separate the effect of the variable from the driving force. There are no reports in the literature which investigate FO operating variables on high osmotic pressure feeds (greater than 55 bar) at similar osmotic gradients. 

This study demonstrates the feasibility of using FO in two waste streams: a raffinate stream from a solvent extraction operation aiming at extracting gallium and a mine water sample from a gold mine. The end goal was to identify the impact of select FO operating variables (cross-flow velocity, membrane orientation, draw salt) on both the kinetics of water removal and rejection efficiency under equivalent osmotic gradient conditions. 

## 2. Experimental Methods

### 2.1. Materials

Draw solutions of NaCl and MgCl_2_●6H_2_O (99.0%, Fisher Scientific, Ottawa, ON, Canada) were first prepared at 5 mol/kg-H_2_O (molal, m) and were then diluted to the desired concentration. Each 5 m stock solution was verified and adjusted based on density correlations (see Supplemental Information). This was especially important for the magnesium chloride solution preparation, as the hexahydrate salt can have varying waters of hydration. Density was measured with a hydrometer (Fisher Scientific, 11-583D), while the temperature was monitored using a digital thermometer (Fisher Scientific, 06-664-27). 

The membrane used was an asymmetrical cellulose triacetate (CTA) FO membrane (Fluid Technology Solutions Inc., Albany, OR, USA, FTS H_2_O) cut in-house to fit a Sterlitech FO cell with a membrane area of 42 cm^2^. A detailed characterization of this membrane was performed previously by our group (Water Permeability, A, 0.85 L/m^2^/h/bar; Salt Permeabilities: B_NaCl_ 0.65 L/m^2^/h, B_MgCl2_ 0.38 L/m^2^/h; Structural Parameter, S, 280 μm) [14]. This was the only commercial flat sheet FO membrane available at the time of experimentation.

The raffinate was supplied by Neo Performance Materials Inc., Toronto, ON, Canada. The solution pH was 1.68 (Thermo Fisher Scientific, Waltham, MA, USA, Orion VersaStar), which was below the tolerance of the CTA membrane used in the study. Thus, pH was adjusted to 3 by the addition of 30 wt% MgO (Fisher Scientific) slurry under magnetic stirring. MgO was used to prevent gypsum (CaSO_4_•2H_2_O_(s)_) formation. The solution composition after pH adjustment and filtration is shown in Table 1. All compositions were measured via Inductively Coupled Plasma–Optical Emission Spectroscopy (ICP-OES, see Supplemental Information) except for Cl^−^, which was measured with an ion selective electrode (Cole-Parmer, Quebec City, QC, Canada, EW-27504-08). The mine water was supplied by Agnico Eagle Mines Ltd., Toronto, ON, Canada, with composition also shown in Table 1.

The Mixed-Solvent Electrolyte (MSE) framework of the OLI thermodynamic modelling software (version 10.0, OLI Systems Inc., Parsippany, NJ, USA) was used to calculate the osmotic pressures of the waste solutions. To perform the calculation, the solution compositions depicted in Table 1 had to be balanced for electroneutrality and pH. As such, specific concentrations of Na^+^ were added to the virtual composition as proxy for all cations, and Cl^−^ for all anions, respectively, and HCl and NaOH were used to achieve the set pH. Given that these were industrial effluent wastes, there were components which were not able to be accounted for. Furthermore, it was not possible to measure the high Cl^−^ concentration accurately due to interferences from other ions. These limitations led to the initial large charge imbalance which was corrected with Na^+^. Nevertheless, even with charge correction, the calculated densities matched the measured densities within 0.9%, which is below the measurement error of the hydrometer. Feed osmotic pressures in this study were assumed constant throughout due to the relatively short duration of experiments, which was verified by sampling and subsequent analysis in the ICP; only 3–9% deviation occurred, which is below the charge balance error. Since draw solution was adjusted in situ, its osmotic pressures were calculated based on the measured concentration achieved with an empirically fit model to OLI simulation data (see Supplementary Information).

### 2.2. Apparatus and Operation

**Figure 2:**
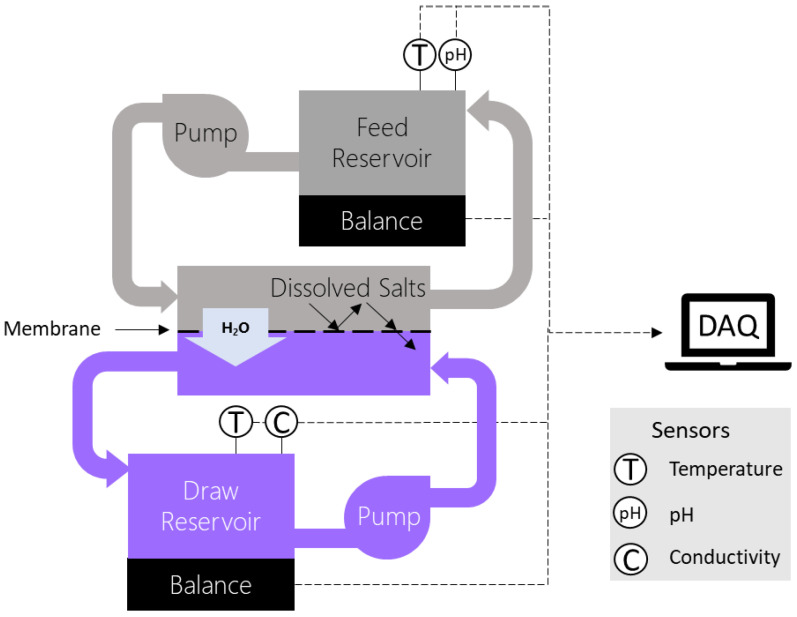
A schematic of the FO circuit configuration. Dashed lines indicate signal connections to the data acquisition system (DAQ).

The FO apparatus shown in Figure 2 consisted of two recirculating loops driven by a multichannel peristaltic pump (7523-20, Cole-Parmer), which passed the feed and draw solutions from their reservoirs (600 mL beakers) through a horizontal membrane housing (Sterlitech Corporation, Auburn, WA, USA, FO Sepa 42) counter-currently. The reservoirs rested atop balances (Mettler Toledo, Columbus, OH, USA, MS4002S) which reported the solution mass at 15 s intervals to a data acquisition system (DAQ). The DAQ provided live water flux readings based on the previous 5 measurements (1 min) of mass measurements. Additionally, connected to the DAQ were: a multichannel pH/conductivity meter (Thermo Fisher Scientific, VSTAR90) with temperature (Thermo Scientific, ORI927007MD), a pH probe (Fisher Scientific, ORI13620631) for the feed reservoir, and a temperature/conductivity probe with a stirrer (Thermo Scientific, ORI013005MD) for the draw side. The DAQ also used a calibration curve for draw solution concentration as a function of conductivity and temperature (see Supplementary Information), and it provided live concentration measurements.

**Figure 3:**
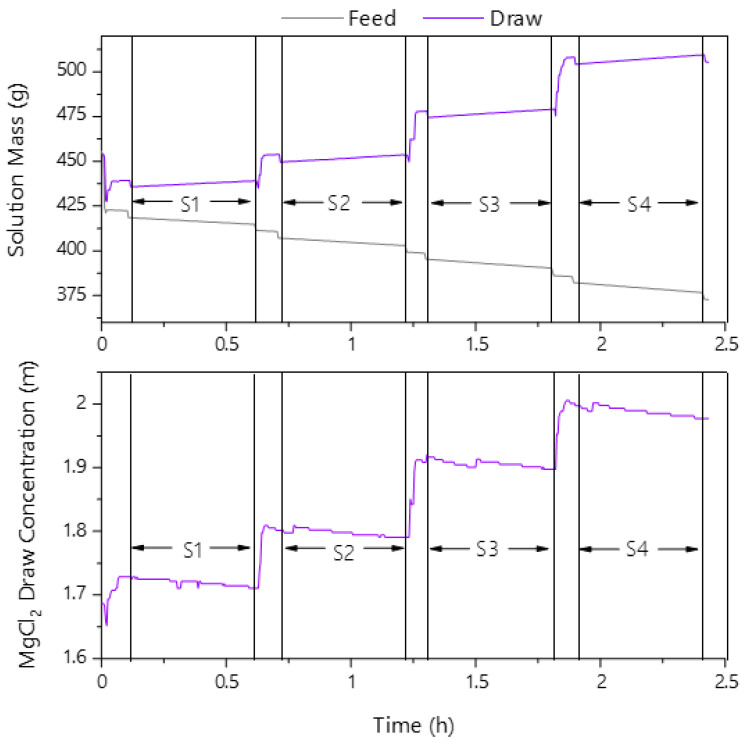
Sample operation data of the feed and draw side online measurements for one trial, stages of operation are labelled S1–S4.

In each trial, four osmotic gradients were tested with each set osmotic gradient referred to as a ‘stage’; the approach was similar to that by Tiafarei et al. [35] and Martin et al. [14]. The operation of the FO apparatus commenced by flushing the system with deionized (DI) water. After draining, the system was primed with a feed and a draw solution, and the pump was then set to run at the desired flow. Once the water flux was stable, a sample was taken from both reservoirs to start the first of four stages of a trial. After 30 min, another sample was taken, marking the end of the stage. Between stages, the draw solution was adjusted to the next target concentration per Table 2 via the addition of 5 molal draw solution or DI water to the previous stage’s draw solution. This procedure was used to control the osmotic pressure gradient, and it maintained constant draw concentrations (±0.01 mol/kg H_2_O) in all stages. Once stabilized, the next sample was taken, and this procedure was repeated for each stage (Figure 3). 

### 2.3. Calculation of FO Metrics

Two FO metrics of performance were tracked in this study: water flux and rejection. Water flux ($J_{w}$) (in L/m^2^/h or LMH) served as the main measure of kinetics. Salt rejection was the measure of separation efficiency. 

The water flux values reported are the average of the water fluxes measured based on the feed and draw mass measurements. Water flux was calculated by linear regression of the mass of each solution versus time in a given stage. The slope of that line was then used in Equation (1) to calculate the water flux [14]:

(1) $$J_{w}=\frac{a_{s}}{A_{mem}\rho_{w}}$$

where $J_{w}$ is the water flux (LMH), $a_{s}$ is the slope of the linear regression line (g/h) of the mass of a solution in a given stage, $A_{mem}$ is the area of the membrane (m^2^), and $\rho_{w}$ is the density of pure water (g/L). 

Salt flux in the direction from the feed to the draw side of the membrane was calculated by the change in the mass of solutes in the draw solution side normalized by the membrane area and the duration the change of mass occurred as shown by Equation (2) [14]:

(2) $$J_{s}=\frac{C_{e}V_{e}-C_{i}V_{i}}{A_{mem}\Delta t_{s}}$$

where $J_{s}$ is the salt flux (mg/m^2^/h), $C_{e}$ and $C_{i}$ are the end and initial concentrations of a stage (mg/L), $V_{e}$ and $V_{i}$ are the end and initial volumes of a stage (L), $A_{mem}$ is the area of the membrane (m^2^), and $\Delta t_{s}$ is the time duration between the salt concentration measurements.

Rejections were calculated by normalizing the salt flux by the water flux followed by normalizing for the average feed side concentration as shown in Equation (3) [36]:

(3) R%=1−JSJwCF,avg×100%

where *R*% is the percent of contaminants rejected by the membrane, $J_{s}$ is the salt flux (mg/m^2^/h), $J_{w}$ is the water flux (LMH), $C_{F,avg}$ is the feed side average contaminant concentration during a given stage (mg/L).

### 2.4. Factorial Design

To minimize the number of experimental trials, a factorial design was used to quantify and obtain an insight into the effects on water flux of single variables (factors) and their 2-factor interactions: two variables multiplied demonstrating synergistic or anti-synergistic effects. Using a factorial design of experiments allows multiple factors to be varied simultaneously while still retaining the ability to calculate the individual impacts on water flux. This design used the methodology presented by Box, Hunter, and Hunter [37] and the terminology therein.

The experiment was designed as a two-level half factorial resolution V ($2_{V}^{5-1}$ = 16 trials). The 5 factors varied between each trial were crossflow velocity, draw salt type, membrane orientation, feed type, and membrane sample. Osmotic pressure was included as an additional factor, but the same 4 levels were tested in each of the trials, so it did not impact the resolution of the design, and it served as replication at different osmotic gradients. Further details on the experimental matrix setup including blocking and confounding techniques are described in the Supplemental Information.

The main factor and 2-factor interaction effects and the corresponding errors were estimated by a multivariate linear regression and analysis of variance (ANOVA). The factors were encoded −1 to 1 (Table 3) to allow the investigation of interactions. The low and high values in the factorial design must be non-zero, otherwise every interaction term would be artificially reduced to zero. For osmotic gradient, the measured value was encoded linearly such that the minimum and maximum values were −1 and 1, respectively. Linear encoding allows the use of measured values to correct for experimental variability instead of the four target values; thus, it represents the actual driving force as accurately as possible. All other factors were binary, and always had a value of −1 or 1.

## 3. Results and Discussion

### 3.1. Water Flux

The mine water showed higher water fluxes than the raffinate with 5.3 LMH versus 2.7 LMH on average, respectively (Table 4). This result was expected, as the raffinate was more concentrated than the mine water, and so, CP was expected to be more severe overall. In the preliminary analysis of effects (see Supplemental Information), membrane sample was not significant at 95% confidence, and it was eliminated from further analysis. This means that membrane heterogeneity was not present. No fouling was observed throughout the study.

**Figure 4:**
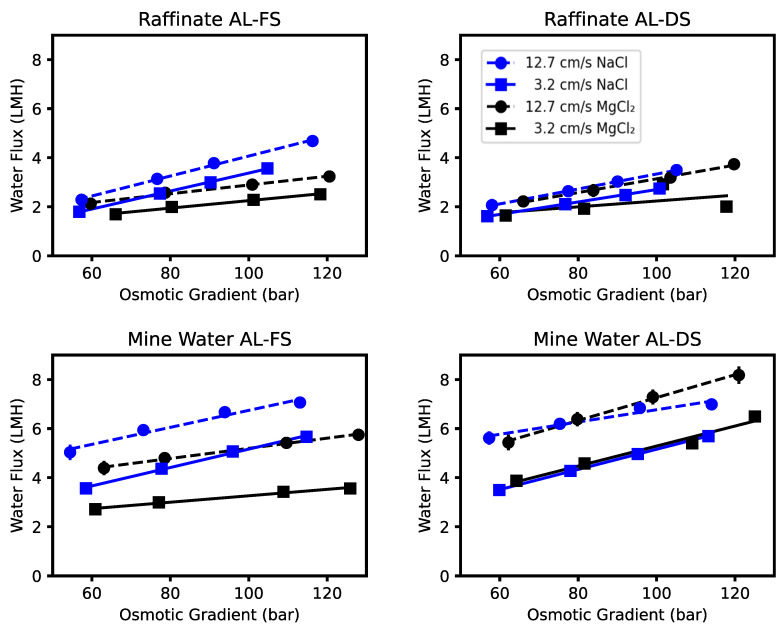
Water flux as a function of osmotic gradient for each feed solution and orientation combination.

Figure 4 shows that water fluxes measured were within what was observed in the literature with other FO operations on industrial effluents. Dou et al. [20] studied the use of FO on a vanadium leaching solution, where they achieved fluxes up to 14 LMH with the same CTA membrane used in this work. Notably, they used saturated NaCl as a draw solution to achieve this, which provided a higher osmotic gradient than what was used in the current work. In a review by Mahto et al. [19], a variety of industrial feeds mostly from oil and gas refining showed fluxes ranging from 2.0 to 9.0 LMH. In another work on drilling mud and oil and gas wastewater, FO operation saw fluxes of ~2–14 LMH [26]. This large range was because neither the feed nor draw solution were controlled throughout their experiments, leading to a dynamically decreasing water flux. In contrast, the draw solutions in this work were controlled to maintain consistent and similar osmotic gradients.

### 3.2. Osmotic Gradient

On average, increasing the osmotic gradient from 54 bar to 128 bar (Δ74 bar) resulted in a flux increase of 1.9 ± 0.4 LMH (67%) and 2.3 ± 0.4 LMH (43%) for the raffinate and mine water, respectively. The water flux results in Figure 4 show a linear response with osmotic gradient. This matches what is expected from the equation [36]:

(4) $$J_{w}=A\Delta \pi_{mem}$$

where A is the membrane water permeability constant (LMH/bar) and $\Delta \pi_{mem}$ is the osmotic gradient over the membrane. The change in water flux relative to the change in osmotic gradient represents 0.026 LMH/bar and 0.031 LMH/bar for the raffinate and mine water, respectively. Compared to the water permeability of 0.851 LMH/bar, this demonstrates CP limitation. Furthermore, osmotic gradient had the largest impact of any factor studied; however, the osmotic gradient was the direct driving force, so it will have the largest impact on energy consumption. Most literature studies in FO on real industrial effluents [20,21,22,23,24,25,26,27,28] used total dissolved solids (TDS); feed and draw concentration; or a proxy for concentration, such as permeate volume and concentration factor, which, while correlated with osmotic gradient, are not directly comparable. Consequently, there is limited ability to compare with works using industrial effluents in a meaningful way, but we encourage future work in this space to provide osmotic gradient estimates to improve this.

### 3.3. Crossflow Velocity

An increase in feed and draw crossflow velocity from 3.2 cm/s to 12.7 cm/s resulted in an average flux increase of 0.7 ± 0.2 LMH (24%) and 1.8 ± 0.2 LMH (34%) for the raffinate and mine water, respectively. This result was expected, as increasing crossflow velocity decreases external concentration polarization (ECP), as shown in multiple works [13,38]. Since the water flux improvement occurred in both membrane orientations, it further suggests this impact is external to the membrane support or active layer (Figure 4), thus confirming this effect is caused by a reduction in ECP. What was surprising was the magnitude of the velocity impact when compared with the other factors. Our earlier work at lower velocities (1.9 and 2.9 cm/s) on the raffinate did not show significant variation [11]. The difference in impacts between the two wastes demonstrates that the raffinate is impacted more by other forms of concentration polarization. 

### 3.4. Draw Salt

**Figure 5:**
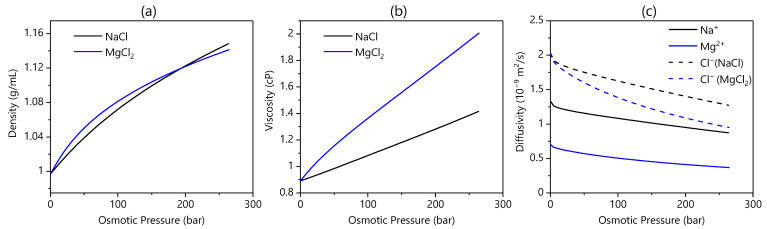
Density (**a**), viscosity (**b**), and ion diffusivities (**c**) as a function of osmotic pressure, calculated from OLI Studio V10.

Changing the draw salt from MgCl_2_ to NaCl caused the flux to increase in AL-FS orientation only (Figure 4). Since this study used equivalent bulk osmotic pressure gradients, the water flux increase demonstrates that NaCl must provide lower CP than MgCl_2_ in AL-FS mode but not AL-DS mode; given this interaction with orientation, the draw salt choice must impact ICP. At equivalent osmotic pressures, MgCl_2_ draw solutions are: more dense, more viscous, and have a lower ion diffusivity than NaCl draw solutions (Figure 5) [39,40]. These properties of MgCl_2_ contribute to the higher ICP compared to a NaCl draw case [14]. Thus, unless high osmotic pressures are required, the use of a NaCl draw solution would improve water flux kinetics. 

This result contradicts what other studies concluded [14,23], suggesting that MgCl_2_ instead of NaCl improves water flux. However, these studies were based on similar draw concentrations instead of similar osmotic gradients. Since MgCl_2_ has a bivalent cation and twice the amount of chloride per mole of solute, it has higher osmotic pressure at the same concentration as NaCl. In addition, the energy demand for draw solute recovery should increase more with increasing osmotic pressure than concentration since osmotic pressure is directly related to the chemical potential of water [41]. Thus, testing at similar concentration misrepresents the driving force, and it falsely concludes that MgCl_2_ is the superior draw salt. Our result corrects this by showing that under the same osmotic gradient, NaCl is superior to MgCl_2_ due to lower viscosity and higher diffusivity.

In the high-concentration feeds studied, reverse salt flux could not be quantified since Na and Mg are already present as concentrated contaminants. The ICP-OES is unable to resolve concentration differences with sufficient precision to allow calculation of the very small change in Na or Mg concentrations; however, given the salt permeability of NaCl (B_NaCl_ 0.65 L/m^2^/h) is greater than MgCl_2_ (B_MgCl2_ 0.38 L/m^2^/h), the reverse draw solute flux will be greater for NaCl [14].

### 3.5. Orientation

On average, a 1 ± 0.2 LMH (18%) increase in flux occurred when changing the membrane orientation from AL-FS to AL-DS mode for the Mine Water only; the impact of Raffinate was insignificant at 95% confidence (Table 4). This implies that the Mine Water experienced more ICP than the draw solution at its osmotic equivalent draw concentrations. Given that the Raffinate did not experience the same impact, it shows that as concentrations increase, the difference between draw solution ICP versus feed solution ICP approaches a similar magnitude. Generally, studies which examined the impact of orientation on dilute (less than 2000 ppm) feeds [21,22,42] demonstrated AL-DS having higher water flux than AL-FS mode, but studies which examined this phenomena as the feed concentrates [22,23,24,28,42] showed that both orientations converged to a similar flux; this study supports the latter. Since orientation also plays a role in fouling reversibility due to the porous support able to retain solids easier, this work recommends the use of AL-FS mode, since there is little benefit to water flux from AL-DS mode.

### 3.6. Salt Rejection

**Figure 6:**
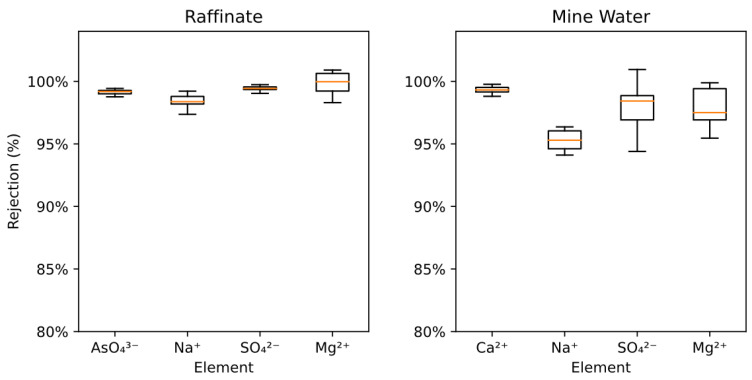
The rejection of concentrated ions by the FO membrane for each feed over all trials. Orange lines are the median, boxes show interquartile range, and the whiskers show the 95% confidence interval.

Across all trials, there are no impacts on rejection efficacy with regard to the operating variables at 95% confidence (*p*-values > 0.05, see Supplemental Information): osmotic gradient (*p*-value 0.81), crossflow velocity (*p*-value 0.148), draw salt (*p*-value 0.156), and orientation (*p*-value 0.079). All rejections were greater than 95%, which is in line with expectations for the FO processes (Figure 6) [13]. Raffinate rejections were slightly higher than Mine Water rejections, demonstrating that more concentrated wastes have a better separation efficacy than more dilute ones. This is due to CP working to hinder the transport of ions from the feed to the active layer surface by lowering the concentration gradient across the active layer; however, while the relative proportion of ions rejected was greater, the absolute flow of contaminants crossing the membrane was still higher for the more concentrated raffinate feed. The lack of correlation with operating variables is beneficial because water flux kinetics can be optimized without the loss of rejection efficacy.

## 4. Conclusions

This study demonstrated the feasibility of water separation by forward osmosis on two highly concentrated and complex effluents from the mining and metals industry. A factorial experimental design was employed, with the aim of quantifying water recovery and salt rejection levels under similar osmotic pressure gradient levels, flow rates, draw salt type, and membrane orientation. It was found that the FO membrane allowed for water fluxes up to 8.2 LMH on the mine water and of 4.1 LMH on the raffinate. This is comparable to the 2–14 LMH seen in the literature with the same type of membrane on other industrial feeds. The lower water fluxes in our experiments versus other FO studies were likely due to a higher concentration polarization effect caused by the greater concentrations of dissolved solids in the raffinate and mine water [19,20,26]. By comparing NaCl vs. MgCl_2_ draw solutions under similar osmotic pressure gradients (i.e., similar bulk thermodynamic driving force), we found that draw salt choice impacts the extent of internal concentration polarization, and in active layer–feed side orientation NaCl draw solute can improve water flux by 30% over MgCl_2_. This falsifies the hypothesis that MgCl_2_ provides improved water flux kinetics over NaCl [14,23]. It was also shown that under concentrated levels of the two feeds tested, a commercial cellulose-tri-acetate FO membrane maintains nominal rejections in excess of 95% for all concentrated ions in the feed solution. This performance was unaffected by the osmotic pressure gradient levels, flow rates, draw salt type, and membrane orientation. Consequently, the operating conditions could be optimized for maximum water flux without compromising the rejection capabilities of the membrane.

## Figures and Tables

**Table 1:** Compositions of tested solutions and OLI simulation charge reconciliations.

	pH Adjusted Raffinate		Mine Water	
pH	3		7	
Osmotic Pressure (Calculated)	125 bar		64 bar	
Composition	mg/L	mol/kgH_2_O	mg/L	mol/kgH_2_O
Na^+^	55,000	2.52	16,000	0.70
Ca^2+^	23	6.1 × 10^−4^	5800	0.15
Mg^2+^	2000	0.09	2000	0.09
Cl^−^	46,000	1.38	50,000	1.44
SO_4_^2−^	63,000	0.70	1800	0.02
AsO_4_^3−^	16,000	0.12	0	0.00
% Charge Imbalance		−14%		−21%
Charge Reconciliation (mol/kgH_2_O Na^+^)		0.44		0.31
pH Reconciliation (mol/kgH_2_O HCl)		0.24		0

**Table 2:** Draw solution concentrations corresponding to a given osmotic gradient Δπ for each feed-draw pair tested.

	Raffinate (125 Bar)		Mine Water (64 Bar)	
Δπ	MgCl_2_	NaCl	MgCl_2_	NaCl
(bar)	(m)	(m)	(m)	(m)
58	1.7	3.3	1.3	2.3
76	1.8	3.6	1.4	2.6
95	1.9	3.8	1.6	2.9
116	2	4.1	1.7	3.2

**Table 3:** The encoding of the factors and blocked variables used to analyze the main factor effects and 2-factor interactions.

	Factors					Blocked Variables	
Encoding	Osmotic Gradient	Crossflow Velocity	Draw Salt	Membrane Orientation		Feed	Membrane Sample
	(bar)	(cm/s)					
−1	54.4	3.2	MgCl_2_	AL-FS		Mine Water	1
+1	128.0	12.7	NaCl	AL-DS		Raffinate	2

**Table 4:** The average change in water flux (Δ LMH) resulting from a factor change from level −1 to 1 with its 95% confidence interval, percent indicates the water flux effect relative to the all trial average for that feed (baseline).

	Factor Level		Raffinate				Mine Water			
Factor	−1	1	Δ LMH			%	Δ LMH			%
Baseline (Average)			2.7				5.3			
Osmotic Gradient	54 bar	128 bar	1.9	±	0.4	67%	2.3	±	0.4	43%
Crossflow Velocity	3.2 cm/s	12.7 cm/s	0.7	±	0.2	24%	1.8	±	0.2	34%
Draw (AL-FS)	MgCl₂	NaCl	0.9	±	0.4	31%	1.6	±	0.5	30%
Draw (AL-DS)	MgCl₂	NaCl	*0.2*	*±*	*0.4*	*8*% *	*−0.2*	*±*	*0.5*	*−4*% *
Orientation	AL-FS	AL-DS	*0.2*	*±*	*0.2*	*8*% *	1.0	±	0.2	18%

* Italics indicate insignificant value at 95% confidence.

## Data Availability

Please contact the authors.

## Supplementary Materials

The following supporting information can be downloaded at: https://www.mdpi.com/article/10.3390/membranes13050501/s1, Table S1: The osmotic pressure model coefficients used for each draw salt. Table S2: The parameters for the molality of solution given a measured density and temperature. Table S3: The parameters for the density of a draw solution given its concentration in mg/L. Table S4: The fitted parameters used with the equation S4 to measure concentration via conductivity in solution. Table S5: The experimental matrix. Each trial has 4 stages with draw salt concentrations designed to maintain a specific osmotic gradient. Table S6: Preliminary analysis of water flux by linear regression and ANOVA, italics indicate insignificant effects removed. Table S7: Summary of multivariate linear regression for rejection impacts.
