# Supplementary material for: Forward Osmosis for Metal Processing Effluents under Similar Osmotic Pressure Gradients
Source: Membranes (Basel). 2023 May 10;13(5):501. doi: 10.3390/membranes13050501 (PMC10222697; doi:10.3390/membranes13050501)
Supplement: Supplementary file 1 [file membranes-13-00501-s001.zip › membranes-2349502-supplementary.pdf]

membranes

Supplemental Information

Forward Osmosis for Metal Processing Effluents under Similar Osmotic Pressure Gradients

Noel Devaere, Vladimiros Papangelakis *

[1] Department of Chemical Engineering and Applied Chemistry, University of Toronto, 200 College Street, Toronto, ON M5S 3E5, Canada
* Correspondence: vladimiros.papangelakis@utoronto.ca

A. Draw Solution Osmotic Pressure

OLI Studio (v10.0) was used to build the osmotic pressure model for the two draw salts used in this study. Osmotic pressure is mainly dependent on the salt solution concentration and temperature. Simulations on draw solutions at various temperatures (15-30 °C) and compositions (0 to 5m) provided the data which was fit by equation S1 for each draw salt.

$$\pi = a_1C^2 + a_2C + a_3T + a_4 \quad (S1)$$

where π is the osmotic pressure in bar, C is the concentration in molal (mol/kgH_2O), and T is the temperature in °C. The coefficients are unique per draw salt type as shown in Table S1.

Table S1. The osmotic pressure model coefficients used for each draw salt.

Draw Salt	a_1 $\left(bar/\frac{mol^2}{kgH_2O^2}\right)$	a_2 $\left(bar/\frac{mol}{kgH_2O}\right)$	a_3 $(bar/°C)$	a_4 (bar)
NaCl	3.96	41.28	0.51	-11.01
$MgCl_2$	41.33	38.00	-0.02	1.04

B. Density Curves

Density was used to verify the concentration of draw solutions and to scale the mass measurements to volumes to calculate the salt fluxes. Two forms of the curve were used in this study, the first was to get the solution molal as a function of density and temperature. Using an equation of the form S2 was found to be the best after analysis of R^2, residual plots, and test/train sampling.

$$C\left(\frac{mol}{kgH_2O}\right) = a_1\rho^2 + a_2\rho + a_3T + a_4 \quad (S2)$$

where C is the molal concentration (mol/kg H2O), ρ is the measured density (g/mL) and T is the temperature (°C). The test train data was sourced from an OLI Studio V10.0 simulation that encompassed compositions from 0 to 5 m and temperatures from 15 to 30 °C to be within normal laboratory fluctuations. Table S2 shows the coefficients for A2 alongside the coefficient of fit.

Table S2. The parameters for the molality of solution given a measured density and temperature.

Draw Salt	a_1 $\left(\frac{mol}{kgH_2O}/\frac{g^2}{mL^2}\right)$	a_2 $\left(\frac{mol}{kgH_2O}/\frac{g}{mL}\right)$	a_3 $\left(\frac{mol}{kgH_2O}/°C\right)$	a_4 $\left(\frac{mol}{kgH_2O}\right)$
NaCl	33.6	−43.3	1.38×10^{-2}	9.43
$MgCl_2$	14.3	−16.0	6.81×10^{-3}	1.55

Further an equation of the form S3 was used to acquire density from a measured cation composition (mg/L) of draw solution.

$$\rho\left(\frac{g}{mL}\right) = a_1C^2 + a_2C + a_3T + a_4 \quad (S3)$$

where ρ is the density (g/mL), C is the concentration of the cation (mg/L) and T is the temperature of the solution (°C). The test train data was sourced from an OLI Studio V10.0 simulation that encompassed compositions from 0 to 275,000 mg/L and temperatures from 15 to 30 °C to be within normal laboratory fluctuations. Table S3 shows the coefficients for S3 alongside their coefficient of fit.

Table S3. The parameters for the density of a draw solution given its concentration in mg/L.

Draw Salt	a_1 $\left(\frac{g}{mL}/\frac{mg^2}{L^2}\right)$	a_2 $\left(\frac{g}{mL}/\frac{mg}{L}\right)$	a_3 $\left(\frac{g}{mL}/°C\right)$	a_4 $\left(\frac{g}{mL}\right)$
NaCl	-1.13×10^{-12}	1.77×10^{-6}	4.16×10^{-4}	1.01
$MgCl_2$	-2.54×10^{-12}	3.04×10^{-6}	-3.19×10^{-4}	1.01

C. Conductivity Calibration Curves

To calibrate the conductivity meter, 6 solutions of known concentration of the draw solution were prepared and their concentrations verified using the density correlations. Calibration involved immersing the conductivity meter in a jacketed sample vial containing 40 mL of the known draw solution while varying the temperature. The conductivity meter reported conductivity and temperature of the sample vial while a second temperature probe in the jacket also recorded during the calibration. The jacket temperature was adjusted to a maximum temperature difference of 2°C via the addition of ice or hot water. The conductivity meter was then varied to a maximum of 30°C and brought down to a temperature of 15°C. The solution crossed each temperature twice, once increasing and once decreasing. The slow temperature change and bi-directional temperature adjustment was used to minimize the effect of hysteresis in the calibration as conductivity responds faster to temperature than the temperature sensor on the conductivity probe.

A randomized 50/50 test train split was used to calibrate the data to the model presented in S4.

$$C\left(\frac{mol}{kgH_2O}\right) = a_1S^2 + a_2S + a_3T^2 + a_4T + a_5ST + a_6 \qquad (S4)$$

where C is the concentration in mol/kg H_2O, S is the conductivity in mS/cm and T is the temperature in °C. The coefficients for each draw salt and their coefficients of fit for each draw salt type are given in Table S4.

Table S4. The fitted parameters used with the equation S4 to measure concentration via conductivity in solution.

Draw Salt	a_1 $\left(\frac{mol}{kgH_2O}/\frac{mS^2}{cm^2}\right)$	a_2 $\left(\frac{mol}{kgH_2O}/\frac{mS}{cm}\right)$	a_3 $\left(\frac{mol}{kgH_2O}/°C^2\right)$	a_4 $\left(\frac{mol}{kgH_2O}/°C\right)$	a_5 $\left(\frac{mol}{kgH_2O}/\frac{mS}{cm}°C\right)$	a_6 $\frac{mol}{kgH_2O}$
NaCl	7.41×10^{-5}	2.10×10^{-2}	1.49×10^{-3}	-2.83×10^{-2}	-8.17×10^{-4}	1.01×10^{-1}
$MgCl_2$	3.97×10^{-4}	-1.17×10^{-2}	4.71×10^{-3}	8.34×10^{-2}	-2.86×10^{-3}	2.66×10^{-1}

D. Inductively Coupled Plasma – Optical Emission Spectroscopy

Inductively Coupled Plasma – Optical Emission Spectroscopy (Agilent Technologies, Santa Clara, CA, USA, 700 Series ICP-OES) was the analytical technique used to measure the concentrations of the feed and draw solutions for the purpose of calculating salt fluxes. Due to the highly concentrated nature of both the feed and draw solution, the samples were diluted to mg/L (ppm) levels, to fit within the instrument calibration range. This work used a serial dilution scheme of 10 and 2500 times dilutions to measure the contaminants within the standards' range of 0-20 ppm. The ICP-OES was calibrated using a custom mixed standard (Inorganic Ventures, via Delta Scientific).

When using ICP-OES measurements from complex and concentrated systems, each measurement occurs on a wavelength that is subject to interferences from other elements present in solution that do not match the standard composition. This causes offsets in some of the elemental measurements on a given wavelength. To account for this, more than one wavelength was used for each element. Each element-wavelength was carried through the calculations, and only at the end (IE once the salt flux / rejection had been calculated) was the median measurement reported as the final number. This allowed the exploitation of the difference terms in the formulae to cancel out some of the offsets that the wavelengths experienced and provide more precise results.

E. Design of Experiments

The experiment was designed as a two-level half factorial resolution V design (2_V^{5-1}, 16 trials) as shown in **Table S5**. The 3 factors varied between each trial were crossflow velocity, draw salt, and membrane orientation. The experiment was blocked to account for the different feeds and membrane variation. Blocking was done by assigning feed and membrane sample as two additional factors. By doing this, if significant variation arose from the membrane or feed type the effect would be quantifiable and independent from the rest of the effects. If no effect was present, the factor can be removed, and the experimental matrix will be the same as if it was unblocked. In doing this, one main factor had to be confounded with a 4-factor interaction term; the 4-factor confounding makes this a resolution V design since 5 variables were involved in the confounding. Membrane orientation was chosen to be confounded since it was expected to have the least effect on water flux owing to the concentrated nature of both the feed and draw streams. Osmotic pressure is considered as an additional factor in the analysis, but the same 4 levels were tested in each of the trials, so it does not impact the resolution of the design which only depends on factors that vary between trials. As such it acts as an experimental replication at different levels of osmotic gradient.

Table S5. The experimental matrix. Each trial has 4 stages with draw salt concentrations designed to maintain a specific osmotic gradient.

	Factors			Draw Concentration (m) for Osmotic Gradient				Blocked Variable	
Trial	Flow Rate (mL/min)	Draw Salt	Membrane Orientation	57 atm	75 atm	94 atm	114 atm	Feed	Membrane Sample
1	400	$MgCl_2$	AL-FS	1.7	1.8	1.9	2	Raffinate	1
2	100	$MgCl_2$	AL-DS	1.7	1.8	1.9	2	Raffinate	1
3	400	NaCl	AL-DS	3.3	3.6	3.8	4.1	Raffinate	1
4	100	NaCl	AL-FS	3.3	3.6	3.8	4.1	Raffinate	1
5	400	$MgCl_2$	AL-DS	1.3	1.4	1.6	1.7	Mine Water	1
6	100	$MgCl_2$	AL-FS	1.3	1.4	1.6	1.7	Mine Water	1
7	400	NaCl	AL-FS	2.3	2.6	2.9	3.2	Mine Water	1
8	100	NaCl	AL-DS	2.3	2.6	2.9	3.2	Mine Water	1
9	400	$MgCl_2$	AL-DS	1.7	1.8	1.9	2	Raffinate	2
10	100	$MgCl_2$	AL-FS	1.7	1.8	1.9	2	Raffinate	2
11	400	NaCl	AL-FS	3.3	3.6	3.8	4.1	Raffinate	2
12	100	NaCl	AL-DS	3.3	3.6	3.8	4.1	Raffinate	2
13	400	$MgCl_2$	AL-FS	1.3	1.4	1.6	1.7	Mine Water	2
14	100	$MgCl_2$	AL-DS	1.3	1.4	1.6	1.7	Mine Water	2
15	400	NaCl	AL-DS	2.3	2.6	2.9	3.2	Mine Water	2
16	100	NaCl	AL-FS	2.3	2.6	2.9	3.2	Mine Water	2

F. Preliminary Water Flux Analysis of Variance (ANOVA)

The preliminary analysis was used to screen for the impacts of variability caused by membrane variation, temperature, and draw solution hysteresis (increasing versus decreasing the draw solution concentration) is shown below in Table S6. Temperature, membrane sample and draw adjustment hysteresis were eliminated as their effects at 95% confidence demonstrated no statistically significant impact. After removing those three variables, the remaining factors were significant at 95% confidence, and their results appear summarized in Table 4.

Table S6. Preliminary analysis of water flux by linear regression and ANOVA, italics indicate insignificant effects removed.

Factor	Coefficient	Standard Error	t	P>|t|	Confidence Interval Lower 95%	Upper 95%
Intercept	3.95	0.093	42.303	0	3.765	4.139
Osmotic Gradient	1.03	0.136	7.616	0	0.761	1.304
Flow	0.70	0.098	7.111	0	0.5	0.892
Draw Salt	0.21	0.098	2.123	0.038	0.012	0.403
Feed	-1.30	0.08	-16.221	0	-1.456	-1.136
Membrane Orientation	-0.17	0.089	-1.938	0.058	-0.349	0.006
Temperature	*-0.34*	*0.263*	*-1.29*	*0.202*	*-0.866*	*0.188*
Membrane Sample	*0.04*	*0.081*	*0.482*	*0.631*	*-0.123*	*0.202*
Draw Adjustment Hysteresis	*-0.14*	*0.102*	*-1.406*	*0.165*	*-0.348*	*0.061*

Italics indicate insignificant value at 95% confidence

G. Rejection Linear Regression and Factor Significance

The rejections were analyzed by multivariate linear regression. A binary factor for each element was used as part of the regression which had a value of 1 or 0 to encode the element type into a numerical form; this element encoding takes the place of the constant in the regression. The resulting regression coefficients are shown in Table S7. Only the element type and the feed type are significant (p-value < 0.05).

Table S7. Summary of multivariate linear regression for rejection impacts

Factor/Block	Coefficients	Standard Error	t-stat	p-value	Confidence Interval Lower 95%	Upper 95%
Osmotic Gradient	*0.03*	*0.132*	*0.241*	*0.810*	*-0.229*	*0.293*
Crossflow Velocity	*0.11*	*0.077*	*1.453*	*0.148*	*-0.040*	*0.263*
Draw	*-0.13*	*0.094*	*-1.425*	*0.156*	*-0.318*	*0.051*
Orientation	*0.14*	*0.077*	*1.767*	*0.079*	*-0.016*	*0.287*
Feed	1.03	0.095	10.801	0.000	0.839	1.214
As	98.11	0.208	472.418	0.000	97.701	98.521
Ca	100.37	0.208	483.319	0.000	99.960	100.780
Mg	98.98	0.222	445.579	0.000	98.539	99.416
Na	96.68	0.209	462.272	0.000	96.264	97.089
S	98.62	0.132	747.541	0.000	98.361	98.882

Italics indicate insignificant value at 95% confidence
